# Special Issue: Genetic Basis of Phenotypic Variation in Drosophila and Other Insects

**DOI:** 10.3390/genes12081212

**Published:** 2021-08-05

**Authors:** J. Spencer Johnston, Carl E. Hjelmen

**Affiliations:** 1 Department of Entomology, Texas A&M University, College Station, TX 77843, USA; 2 Department of Biology, Texas A&M University, College Station, TX 77843, USA

Next-generation sequencing provides a nearly complete genomic sequence for model and non-model species alike; however, this wealth of sequence data includes no road map. How genetic sequencing generates the observed diversity of living forms remains largely unanswered. The complexity of this roadmap is to be expected; interactions among coding and non-coding sequences can change the levels and timing of expression, creating diversity. Novel phenotypes may emerge from these interactions, and the diversity generated may be further altered by environmental interactions. The 10 articles in this Special Issue converge on these interactions. Tools to untangle the sources of diversity are described. Both historic and novel phenotypes are addressed and discussed. Within *Drosophila,* articles investigate sleep, decision making, immune defense, desiccation resistance, and partner choice. Outside of *Drosophila*, works include predatory behavior in fireflies, host shift in seed beetles, and species diversity in tiger beetles.

The multiple authors of the Special Issue are among the emerging leaders in this effort, and they share their contributions toward generating a road map connecting the gene networks and phenotypic diversity created by genetic and environmental change. McKinley and Lower [1] describe the unique challenges of and the many opportunities for connecting phenotype and genotype in non-model organisms with their often complex and intriguing behaviors. They use comparative transcriptomics to examine divergent behavior phenotypes in predatory and non-predatory species of fireflies in the genus *Photinus*. Rather than looking for differentially regulated genes in the two phenotypic classes, the authors identify genes that show evidence of positive selection. Nine gene families were identified under positive selection in the predatory versus non-predatory *Photuris* comparison, including genes involved in digestion, detoxification, vision, reproduction, and neural processes. These results generate intriguing hypotheses about the genetic basis for insect behavior and highlight the utility of comparative transcriptomic tools to investigate complex behaviors in non-model systems. Burns, Cavallaro, and Saltz [2] describe behavioral assays that compare decision making in *Drosophila sechellia* and *D. simulans*—two recently diverged species that differ substantially in habitat breadth, environmental predictability, and variability. They showed that, as hypothesized, environmental unpredictability was associated with higher decision-making accuracy. Unexpected was that environmental unpredictability was not associated with exploratory behavior, and equally unexpected was a strong difference between the sexes that extended to “handedness”. Females exhibited lower habitat choice accuracy when the preferred substrate was on the right. This study takes a valuable early step investigating the environmental factors and ultimately the genetics influencing the evolution of decision making. Rêgo et al. [3] address the genetic basis of adaptation, including constraints on parallel evolution. Using evolve and resequence, genotype–phenotype association mapping, and an analysis of differential gene expression, they tease out the multifaceted nature of adaptation to a novel host environment. Adding a twist to the search for a road map, they move from a genotype–phenotype map to a genotype–phenotype–fitness map. They find considerable parallelism in allele frequency change, yet with limited parallelism in genotype–phenotype associations for weight and development time. They suggest that selection for survival drove genetic change in adaptation to a new host rather than change in the genetic architecture of performance traits or differential gene expression. Smith and Macdonald [4] measured a battery of sleep phenotypes in >750 genotypes derived from a multiparental mapping panel and identified several, with modest-effect QTL contributing to natural variation for sleep. The list of possible candidate causative sleep loci was narrowed by comparisons with transcriptomic eQTLs from the same mapping panel. Using nervous system-specific RNAi, the sleep-related role of Dopa decarboxylase, dyschronic, and timeless genes was validated—all strong candidates to harbor causative, regulatory variation contributing to sleep. Duran et al. [5] argue that the relationship of genotype to phenotype has immediate taxonomic and conservation importance. They link phenotype and genotype by examining the population genetic structure of phenotypic variants collected in variable environments. Using mitochondrial sequence and a variety of nuclear markers from described subspecies and color forms of the *Cicindelidia* complex of tiger beetles, they create a mitochondrial genealogy, a multilocus nuclear phylogeny, a principle component analysis display, and a structure figure showing the genetic makeup representative of the collected phenotypic diversity. These show discordant mtDNA and nuclear marker patterns; phenotypic variation below the species level was not associated with patterns of genetic structuring. Geographically associated life history traits (i.e., seasonality and elevational preferences) explained the unexpected existence of an undiscovered cryptic species. Johnston, Zapalac, and Hjelmen [6] report differences in DNA underreplication and ploidy in the indirect flight muscles of *Drosophila*. The majority of underreplicated nuclei in the thorax of *Drosophila* are in the dorsal longitudinal muscles (DLM), where fully half of the DNA replication in the DLM nuclei stalled at S phase between the unreplicated G0 and the fully replicated G1. A lesser number of nuclei are in the dorsal ventral flight muscle, where replication stalls earlier (less DNA is replicated), and the endocycle is initiated. These tissue differences were unreported to date and provide a new tool to study heterochromatin, underreplication, and endocycle control. Chapman, Dowell, Chan, and Unckless [7] explore the genetic basis of variation contributing to defense against a pathogenic bacterium via a comparison among inbred lines of the *Drosophila* Genetics Reference Panel. They identified six genes associated with survival, yet, surprisingly, none were canonical genes of the innate immune system. RNAi knock down of the candidate genes confirmed a role for two of them and suggested a role for a third. Importantly, the inference made is that “genetic causes of variation in immune defense differ for different pathogens”. Hjelmen et al. [8] score genome size in two dipteran species selected for phenotypic diversity in body size and development rate. They ask if this direct selection created anticipated strain-specific changes in overall genome size. Although cell size and replication rate are strongly correlated with genome size, increased genome size was observed only for a subset of large body size lines that were subject to a population size bottleneck. Equally unexpected was that strong divergent selection for development rate produced convergence on an intermediate genome size in the fast and slow developing lines. Davis and Moyle [9] compare gene expression in three species that differ in desiccation resistance. Their goal was to determine the number of genes, and the level of expression of the genes, that confer resistance to desiccation. Surprisingly, the species with the highest desiccation resistance had the fewest genes with plastic expression changes. They conclude that species-specific expression difference is likely based on a limited set of loci with either constitutive or plastic gene expression responses. Differences in desiccation resistance was not due to broad genome-wide gene expression differences. Intriguing potential sex-specific mechanisms of desiccation resistance are also discussed. Sato et al. [10] harness the power of *Drosophila melanogaster* genetics to address an area where little is known, viz. the molecular genetic basis of species-specific gain or loss of a discrete behavioral action arising from a change in gene expression. They trace individual neural circuits and show gene expression patterns responsible for the courtship “song” of *D. melanogaster* and of nuptial gift transfer that is unique to *D. subobscura*. Their detailed description of neural circuitry is followed by a description of the new tools that they predict will result in explosive development of the field, and they predict that the explosion will occur in model and non-model insects in the very near future.

Overall, the papers in this Special Issue provide examples of the variety of phenotypes amenable to studies linking phenotype to the genotypes. All stress that high-quality genomes and transcriptomes are increasingly available. All show that combinations of existing and new analytics applied to this wealth of data allow the field to move forward in discovering this genetic “road-map”, which leads to the diversity we have long observed with the naked eye.
